# The Influence of Antidepressants on the Immune System

**DOI:** 10.1007/s00005-019-00543-8

**Published:** 2019-04-29

**Authors:** Łukasz P. Szałach, Katarzyna A. Lisowska, Wiesław J. Cubała

**Affiliations:** 1 Department of Physiopathology, Medical University of Gdańsk, Dębinki 7, 80-210 Gdańsk, Poland; 2 Department of Adult Psychiatry, Medical University of Gdańsk, Gdańsk, Poland

**Keywords:** Depression, Antidepressants, Lymphocytes, Cytokines, Proliferation, Apoptosis

## Abstract

Depression is one of the most frequently diagnosed condition in psychiatry. Despite the availability of many preparations, over 30% of treated patients do not achieve remission. Recently the emphasis is put on the contribution of the body’s inflammatory response as one of the causes of depression. The interactions between nervous and immune systems are the main issue addressed by psychoneuroimmunology. In patients suffering from depression changes in the plasma concentrations of cytokines and in the number and level of activation of immune cells has been found. Attention is paid to the high levels of pro-inflammatory cytokines, the prevalence of Th1 responses to Th2, weakening of NK cell cytotoxicity and changes in lymphocyte proliferation and apoptosis. A number of studies focus on influence of antidepressants and non-standard methods of depression treatment, such as ketamine infusion, on patients’ immunology. Many of them seem to regulate the immune responses. The study results encourage to look for new ways to treat depression with immunomodulatory drugs. In this article authors present the current knowledge about immune system changes accompanying depression as well as the study results showing the influence of drugs on the immune system, especially in the context of reducing the symptoms of depression.

## Introduction

Depression is a condition that occurs in the course of many psychiatric disorders. It can be caused by various factors and have varying severity and duration. Using the term “depression” we usually mean a major depressive episode during the recurrent depressive disorder or major depressive disorder. A depressive episode can be also a component of bipolar disease in which depressive episodes are intertwined with manic episodes. Typical symptoms of depression include depressive mood, anhedonia, sleep changes, psychomotor retardation, decreased appetite and libido, weight loss, feeling of worthlessness as well as suicidal thoughts and suicide attempts, although not all of them must be present for the diagnosis of depression. To meet the criteria of a depressive episode (both according to ICD-10 and DSM-V), the symptoms should last for a minimum of 2 weeks for most of the day. These symptoms may be accompanied by anxiety and psychotic symptoms, such as delusions (most often of guilt or sin) and hallucinations (American Psychiatric Association 2013; World Health Organization 2016).

Depression as a syndrome is the body’s response to a variety of genetic, psychological, environmental and biological factors (Murawiec and Wierzbiński 2016). Nowadays attention is paid to the role of inflammation resulting from the activation of the immune system in the course of depression, especially in its drug-resistant form. Also, it has been proven that antidepressants modulate immune responses thus affecting the activation, proliferation and survival of leukocytes.

**Fig. 1:**
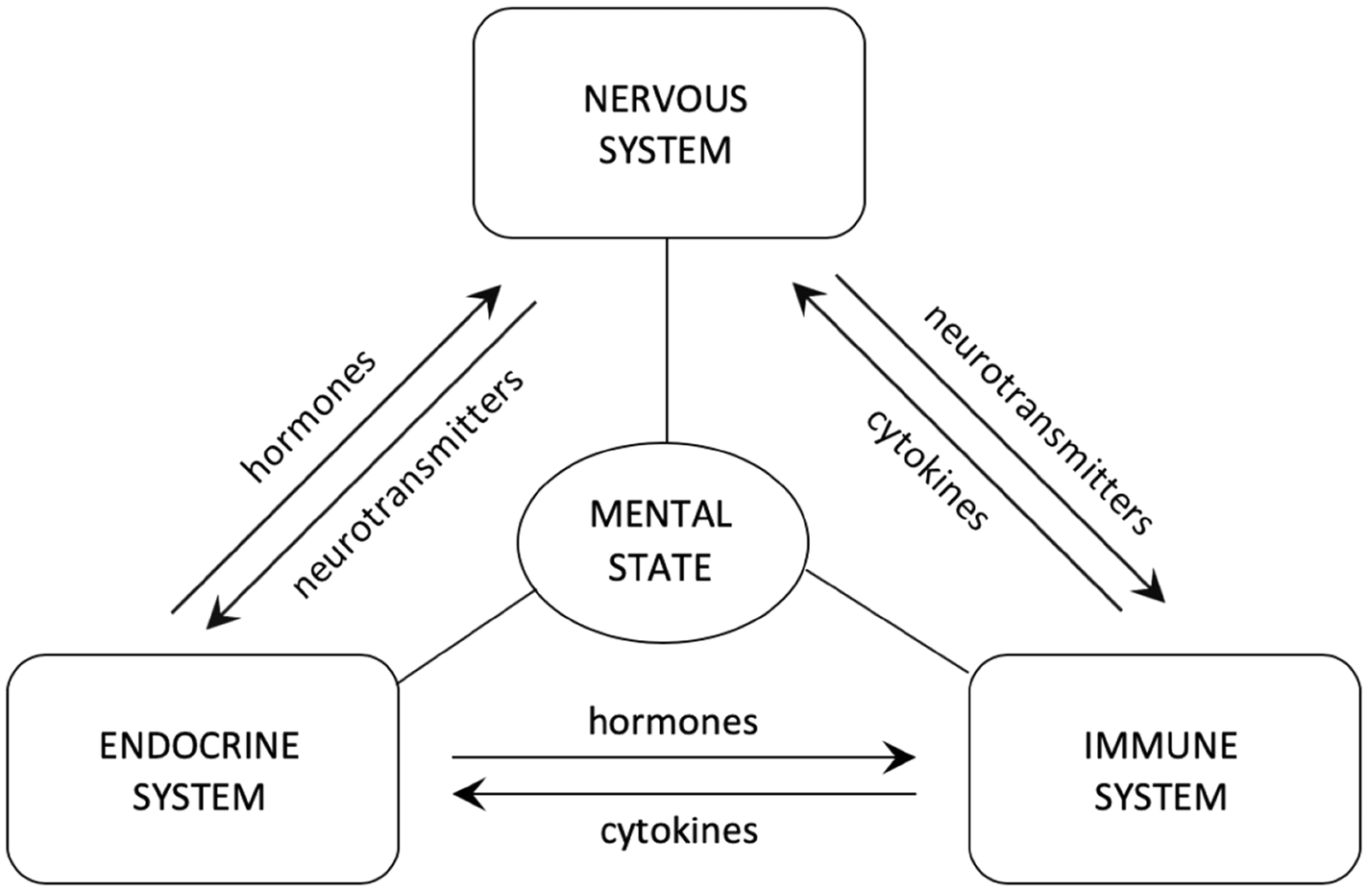
The interactions between nervous, endocrine and immune systems modulated by secreted hormones, neurotransmitters and cytokines

Psychoneuroimmunology is a field of study based on the concept of mutual interaction between nervous, endocrine and immune systems taking into account the influence of mental state on patient’s immunity (Pariante 2015) (Fig. 1). It has been scientifically proven that the function of lymphatic organs and cells of the immune system can be regulated by the nervous system with the help of hormones, secreted mainly by the pituitary gland, and by neurotransmitters, among others, noradrenaline, acetylcholine released directly from the axons of cells of the autonomic nervous system (Talbot et al. 2016). Lymphocytes and macrophages are also known to express on their surface a number of receptors for neurotransmitters like noradrenaline, acetylcholine, dopamine, serotonin, gamma-aminobutyric acid, endorphins and hormones such as corticotropin (CRH), allowing them to respond to signals sent by nervous system (Rybakowski 2002). In response to the stimulation of receptors with the aforementioned substances, a number of processes in immune cells are activated, including the production of cytokines secreted into the bloodstream allowing their systemic action, and hormones, mainly pituitary, such as corticotropin (ACTH), thyrotropin (TSH) or endorphins, mainly acting locally as auto- and paracrine hormones (Pállinger and Csaba 2008).

Cytokines of immune cells are secreted both systemically and locally within the central nervous system (CNS). Some of them, such as interleukin (IL)-6, delivered by the bloodstream can pass through the blood–brain barrier (BBB) (Banks et al. 1994). They also affect indirectly the inflammatory process within the central nervous system by cerebral endothelium stimulation for the expression of adhesion molecules with co-stimulatory properties, such as intercellular adhesion molecule 1 and vascular cell adhesion protein 1, and the major histocompatibility complex class II with interferon (IFN)-γ, so that T lymphocytes specific for the brain antigens may be activated to cross BBB and stimulate the inflammation. Additionally, both neurons and glial cells also can produce and secrete cytokines locally (Stokłosa 2017).

Knowledge about mechanisms of anti-inflammatory and pro-inflammatory processes, activity of immune cells and their mutual relations with the nervous and endocrine systems may help to understand the role of immune system in affective disorders. The purpose of this article is to show that, in addition to the classical effects on the neurotransmitter system in the brain, antidepressants can also modulate the immune responses in patients suffering from depressive disorders, and thus contribute to clinical state improvement in patients during anti-depressive treatment.

## Immune System Dysregulation in Depression

Regulation of immune system activity and chronic inflammation seems to play an important role in the pathogenesis of depression. One of the basic and best-studied mechanisms of interaction between nervous, endocrine and immune systems is their mutual influence during chronic stress or chronic inflammation accompanying some of the patients presenting features of the depressive syndrome.

Stress induces the transmission of nerve signals from the cerebral cortex to the hypothalamus where it stimulates the secretion of CRH, then ACTH by the pituitary gland which results in the release of glucocorticoids from adrenal cortex. Glucocorticoids act in immunosuppressive manner on the immune cells through the inhibition of cytokines production essential for the development of the inflammation, e.g., IL-1, IL-6, tumor necrosis factor (TNF)-α, IL-2 or IFN-γ, simultaneously reducing the T lymphocytes activity (Oppong and Cato 2015). They also favor the recruitment of transcription factors to promoters of genes encoding proteins with anti-inflammatory properties, such as IL-10, I κB, annexin 1 or α-2-microglobulin (Oppong and Cato 2015).

**Fig. 2:**
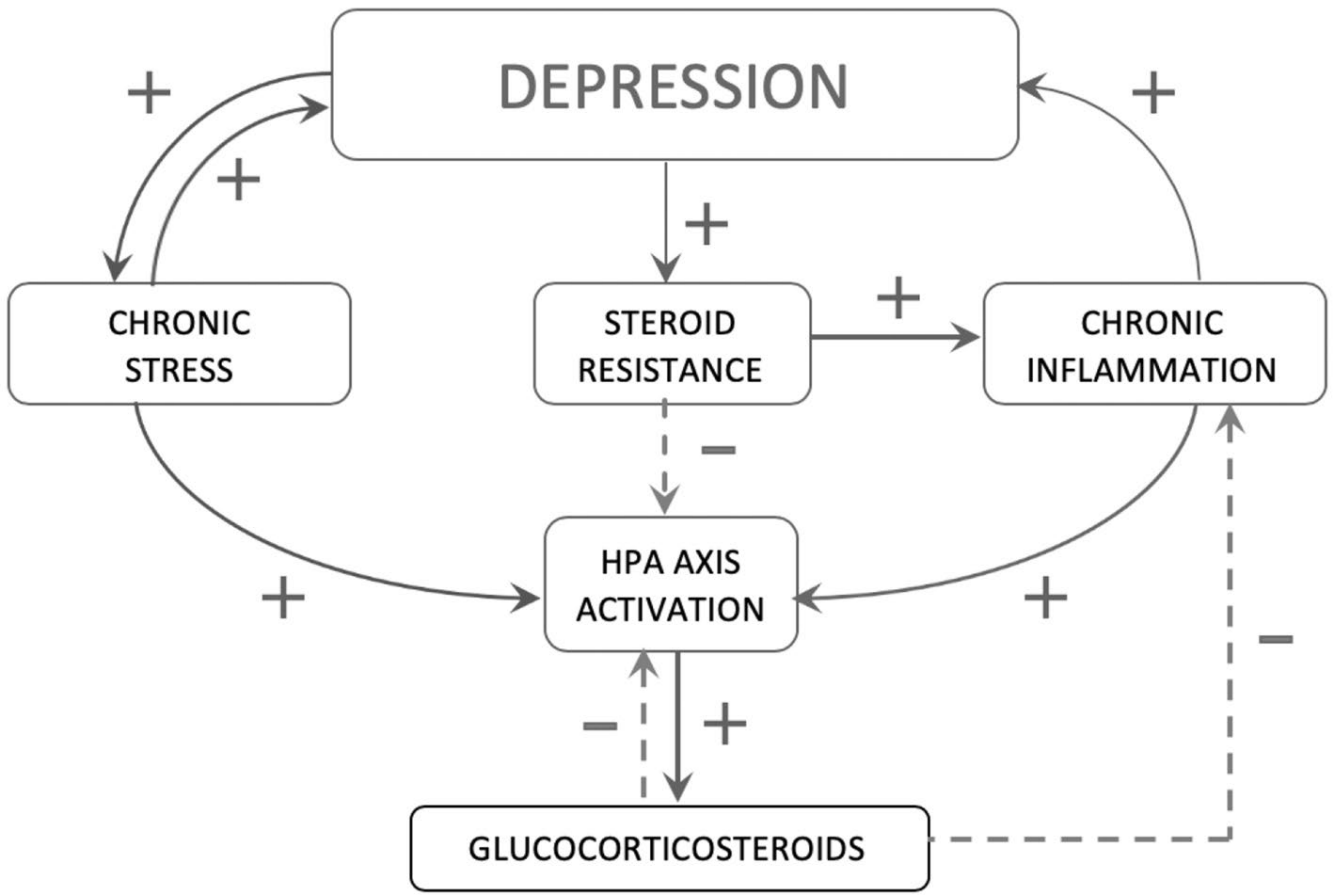
The participation of chronic stress and chronic inflammation in the development of depression and steroid resistance

The CNS can also inhibit the immune responses during chronic inflammation; when locally activated immune cells secrete into the blood stream a number of pro-inflammatory cytokines (IL-1, IL-6, TNF-α), the hypothalamic–pituitary–adrenal (HPA) axis is secondarily stimulated which consequently leads to the secretion of CRH and ACTH. Again, this results in suppression of the immune response through adrenocortical hormones (Hilal-Dandan and Brunton 2014). Unfortunately, in the situation of depressive episode, the immunosuppressive effect of the HPA axis seems to be insufficient to reduce inflammation associated with depression, which may be result of steroid resistance of immune cells or decrease in the threshold of hypothalamic sensitivity to pro-inflammatory cytokines secreted by these cells (Hilal-Dandan and Brunton 2014) (Fig. 2).

**Fig. 3:**
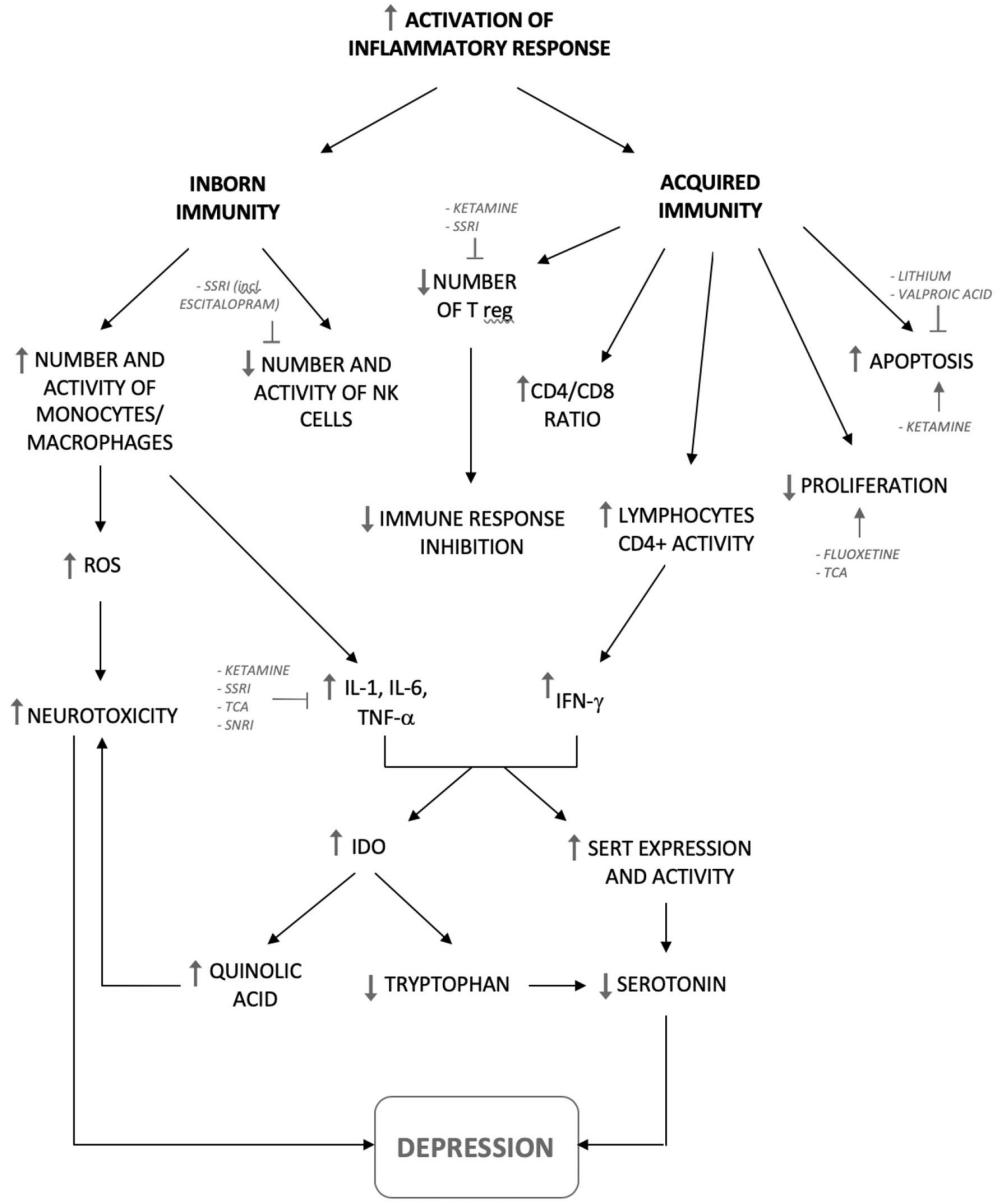
Changes in the immune system in depressive patients with the identification of pathways modified by antidepressants. SERT: serotonin transporter

The inflammatory hypothesis of depression has been described and confirmed in a number of scientific studies. Elevated concentration of cortisol was observed in the blood of patients with the first major depressive episode diagnosed at an early age (Cubała and Landowski 2014, Cubała et al. 2016). It has been demonstrated that concentrations of pro-inflammatory cytokines are elevated in the serum of patients suffering from depressive disorders, mainly IL-1, IL-6, IFN-γ and TNF-α, which has been confirmed in several meta-analyzes (Dowlati et al. 2010, Haapakoski et al. 2015, Schmidt et al. 2014a) (Fig. 3). The increase in the level of pro-inflammatory cytokines was accompanied by increased plasma concentrations of granulocyte–macrophage colony-stimulating factor (Schmidt et al. 2014a) and monocyte chemoattractant protein 1 (Kiraly et al. 2017). Pro-inflammatory cytokines are mainly produced by type 1 T helper (Th1) cells which together with macrophages, dendritic cells and cytotoxic T cells are involved in a cell-mediated immunity essential for the elimination of the intracellular pathogens. Elevated concentrations of other cytokines, such as IL-5, IL-7, IL-8, IL-10, IL-12, IL-13 (Schmidt et al. 2014a) have also been reported in some studies. These cytokines are involved in the development of humoral response against extracellular pathogens supported by Th2 cells. Increased activation of inflammasome NLRP3 (a multi-oligomer responsible for the activation of inflammatory responses) in peripheral blood mononuclear cells (PBMCs) of patients suffering from the depression has been described as well (Alcocer-Gómez et al. 2017). It is also known that IFN-α exogenously administered to treat chronic viral hepatitis C can cause transient symptoms of depression (Bonaccorso et al. 2002). In patients vaccinated against Salmonella typhi depressive symptoms occurred after vaccine administration and were positively correlated with elevated levels of IL-6 (Wright et al. 2005).

Changes of the immune system in patients suffering from depression are also noticeable in the number and ratio of leukocyte populations. An increase in the number of cells involved in the inborn immune responses, i.e., monocytes, macrophages and neutrophils (Demir et al. 2015), as well as increased production of reactive oxygen species (ROS) has been reported (Wei et al. 2015). In turn, a reduction in the number of NK cells, which are responsible for natural cytotoxicity was observed (Patas et al. 2018), accompanied by a simultaneous decrease in their activation compared to healthy people (Stokłosa 2017, Zorrilla et al. 2001). Some authors reported an increase in the ratio of CD4^+^ T (Th) cells to CD8^+^ cytotoxic (Tc) T lymphocytes (Di Rosso et al. 2016; Zorrilla et al. 2001). In addition, T lymphocytes of depressed patients presented significantly reduced expression of chemokine receptors CXCR3 and CCR6 (Patas et al. 2018), which play an important role in the recruitment of leukocytes to places where the inflammatory process is present. Additionally, an increase in the percentage of CD25^+^ cells (activated T cells) (Müller et al. 1993; Patas et al. 2018) with the accompanying decrease in the total number of regulatory T lymphocytes (Treg) (Toben and Baune 2015), which are responsible for suppression of immune responses, within CD4+ T cell population have also been observed. Some authors demonstrated that lymphocytes of depressed patients were also characterized by decreased response to mitogen stimulation in vitro (Darko et al. 1989; Kronfol et al. 1986; Toben and Baune 2015), while study of Schleifer et al. (1999) reported the opposite effect. Moreover, PBMCs of patients were also more prone to apoptosis (programmed cell death) compared to healthy people (Ivanova et al. 2007).

Even though there are animal and human studies suggesting that there can be stress-related B lymphocyte decrements due to high levels of glucocorticoids, both the number of B cells and the level of antibodies in the serum of patients suffering from depression seem to be comparable to those observed in healthy persons (Dubois et al. 2016). Some authors suggest that autoantibodies, such as anti-ribosomal-P and anti-*N*-methyl-d-aspartate receptor antibodies, which are present in patients suffering from autoimmune diseases such as systemic lupus erythematosus might contribute to pathophysiology of depression in that group (Iseme et al. 2014; Postal and Appenzeller 2015). Recently, Ahmetspahic et al. (2018) demonstrated that frequencies of naive B cells as well as regulatory B cells are reduced in severely depressed patients as compared to healthy donors or mildly to moderately depressed patients, which suggests that the humoral response could play a role in the development of inflammation in the course of depression. However, there are still only a few articles about disorder of the humoral response in patients suffering from depression and this topic requires further research.

The changes in the immune system described above may contribute to the development of depression in various ways (Schmidt et al. 2014a). The pro-inflammatory cytokines such as IFN-γ, IL-1 or IL-6 affect the HPA axis by stimulating the secretion of CRH and increasing steroid resistance (Cubała and Landowski 2014), which secondarily stimulates the organism to produce more immunosuppressive glucocorticoids (Pariante and Lightman 2008). Also, cytokines induce production of inducible nitric oxide synthase, which leads to increased synthesis of ROS that cause protein and DNA damage that result in neurotoxicity and neurodegeneration (Inserra et al. 2018). Moreover, NO decreases norepinephrine production (Karolewicz et al. 2001) and inhibits the dopamine transporter, which decreases the availability of inter-synaptic dopamine (Sandor et al. 1995).

The most known neurobiological theory explaining mechanism of depression is monoamine theory, especially the serotonin hypothesis. The serotonin hypothesis of depression postulates that a reduction in serotonin levels may cause depression (Cowen and Browning 2015). Indeed, most of the widely used anti-depressive medications in the medical practice focus on increasing serotonin level in the synaptic cleft, mostly by inhibition of the serotonin reuptake. There are studies showing that immune system may interfere with serotonin system and contribute to the decrease of serotonin levels in CNS. It is postulated that pro-inflammatory cytokines, such as IFN-γ, IL-1 or IL-6, produced by activated immune cells are responsible for triggering pro-depressive effects through the induction of indoleamine 2,3-dioxygenase (IDO), an enzyme involved in the metabolism of tryptophan. IDO activates kynurenine pathway, thus causing depletion of tryptophan, which may be one of the important factors decreasing serotonin concentration in depressed patients. In addition to lower serotonin levels, there is also an increase in neurotoxic metabolites of kynurenine−quinolic acids (Zou 2015). Moreover, pro-inflammatory cytokines also increase the expression and activity of the presynaptic serotonin transporter, which results in more excessive neurotransmitter reuptake from the synaptic space (Chou et al. 2016; Malynn et al. 2013). However, it is important to notice that only 50% of patients respond to the selective serotonin reuptake inhibitors (SSRI) and effective remission occurs less than 30% of the time (Trivedi et al. 2006). In the non-responders group, changing SSRI to other antidepressants that modulate also dopamine and/or norepinephrine levels causes remission in only two-thirds of patients (Stahl 2013). It shows that other mechanisms causing depression, not connected to synaptic serotonin or other monoamines levels, such as dopamine and norepinephrine, must exist.

## Antidepressants and Their Effect on the Immune System

Currently, as mentioned above, the main drugs used for pharmacotherapy of depression are drugs that affect the level of neurotransmitters in the synaptic cleft, such as tricyclic antidepressants (TCA), selective serotonin/serotonin and noradrenaline reuptake inhibitors (SSRI and SNRI) as well as antiepileptic drugs (e.g., lamotrigine, valproic acid) and antipsychotics (including quetiapine, olanzapine, amisulpride). In addition to the known mechanism of action of these drugs on neurotransmitter transporters and pre- and post-synaptic receptors located on the neuronal membrane (serotoninergic, dopaminergic, adrenergic), it is considered that they may have immunomodulatory properties which can contribute to achieving a therapeutic effect in patients with depression through this mechanism as well.

A meta-analysis by Strawbridge based on 35 clinical trials performed in over 20 years has shown that in patients suffering from unipolar depression, who have responded to widely understood antidepressant treatment, serum level of TNF-α and IL-6 significantly decreased (Fig. 3) while no significant change in C-reactive peptide concentration before and after treatment was observed (Strawbridge et al. 2015). Other meta-analysis involving the examination of 22 studies showed that the level of IL-1β in patients’ serum significantly decreased after pharmacotherapy (SSRI, SNRI or TCA), IL-6 level decreased slightly in patients receiving SSRI, while TNF-α levels did not change significantly regardless of the taken medicine (Hannestad et al. 2011). In study of Dahl et al. (2014), which measured peripheral blood cytokine levels before and after 12-week antidepressant therapy in 50 patients, a significant reduction in the level of cytokines IL-6, IL-7, IL-8, IL-10, G-CSF, IFN-γ and IL-1 receptor antagonist was reported. Importantly, the drop in the levels of these cytokines was seen only in patients who demonstrated clinical response to treatment (Dahl et al. 2014). In another randomized trial a decrease in the concentration of pro-inflammatory cytokines was also observed in 73 patients who received sertraline (some of the patient received also transcranial magnetic stimulation therapy). Unfortunately, in this study the decrease also occurred in people taking placebo and was not related to the antidepressant effect (Brunoni et al. 2014). In another study, in which the level of soluble TNF receptor (sTNFR) was measured before and after treatment with sertraline, showed no significant difference in the concentration sTNFR in plasma after sertraline administration (Brunoni et al. 2015).

Antidepressants can influence activity and numbers of immune cells as well but not only that. For example, fluoxetine and mirtazapine have been shown to increase the expression of the serotonin transporter on the surface of T lymphocytes (Peña et al. 2005), which may improve the reuptake of serotonin thus lowering its concentration in the environment of lymphocytes and, consequently, at the same time reduce their ability to proliferate (Ahern 2011). Immunomodulatory properties of fluoxetine have also been reported in animal models and human. In mice treated with fluoxetine a number of T lymphocytes in the peripheral blood was decreased but the CD4^+^/CD8^+^ ratio remained unchanged (Di Rosso et al. 2016). In vitro, a decrease in the proliferative activity of T lymphocytes in patients with depression, who were treated fluoxetine, was also observed (Fazzino et al. 2009). In another study, not only the decrease in the concentration of pro-inflammatory cytokines was observed in 16 patients, after 6 weeks of treatment but also an increased number of Treg cells was observed (Grosse et al. 2016; Himmerich et al. 2010). Schleifer et al. (1999), who demonstrated that in patients with depression T cell response to mitogenic stimulation is significantly increased compared to healthy people, also showed that in patients receiving TCA lymphocyte activity significantly decreases. Several studies showed that pharmacotherapy of depression can also increase the number and cytotoxic activity of NK cells (Mizruchin et al. 1999; Park et al. 2015).

Alcocer-Gómez et al. (2017) examined the expression level of genes encoding elements of NLRP3 inflammasome in mononuclear cells taken from patients receiving antidepressants from the SSRI group (paroxetine), SNRI (venlafaxine, desvenlafaxine), TCA (imipramine, amitriptyline) and patients treated with agomelatine or mianserin, were measured. All of these drugs not only significantly decreased the expression of genes but also reduced inflammasome activity, which decreased of plasma concentrations of IL-1β and IL-18 (Alcocer-Gómez et al. 2017). Changes in the concentration of brain-derived neurotrophic factor (BDNF), a factor responsible for the survival of neurons, stimulating the formation of new nerve cells and synapses, with a broadly defined anti-inflammatory properties, were also observed (Papathanassoglou et al. 2015). Increase in patients’ serum BDNF has been demonstrated as soon as 5 weeks of treatment with sertraline and 6 months when venlafaxine was administered (Matrisciano et al. 2009). Moreover, studies show that the measurement of BDNF in serum may correlate with the level of BDNF in the central nervous system (Klein et al. 2011).

In the recently published work of Pietruczuk et al. (2018) it was demonstrated that T lymphocytes of patients suffering from bipolar disorder treated with lithium or valproate are characterized by decreased proliferative activity and increased susceptibility to apoptosis. The authors also showed that in vitro both drugs have an anti-apoptotic effect but do not affect in any way the ability of lymphocytes to proliferate (Pietruczuk et al. 2018).

Ketamine, an intravenous anesthetic affecting glutamate neurotransmission, which has also been used in the treatment of drug-resistant depression, also modulates the immune response. Presently, there are no studies showing how ketamine can influence the immune system of patients suffering from affective disorders. However, its immunomodulatory effect has been shown in oncological patients and patients in the postoperative period. It seems that low doses of ketamine administered parenterally cause a temporary decrease in TNF-α and IL-6 concentration in the patient’s blood in the postoperative period (Beilin et al. 2007). Studies performed on mononuclear peripheral blood cells collected from oncological patients showed that ketamine may increase the CD4+/CD8+ ratio as well as the percentage of Treg cells (Hou et al. 2016). Meanwhile, studies in healthy subjects have shown that ketamine can inhibit the differentiation of Th2 cells, which are responsible for regulating humoral responses (Gao et al. 2011). It has also been shown that ketamine may exhibit an immunosuppressive effect not only by reducing the pro-inflammatory cytokines synthesis or the ability of the leukocyte to adhesion and migration (Larsen et al. 1998; Kawasaki et al. 2001) but also by inducing apoptosis of T lymphocytes (Braun et al. 2010) and the inhibition of the maturation of dendritic cells responsible for the antigen presentation (Zeng et al. 2011).

## Immunomodulatory Drugs as Antidepressive Treatment?

An additional argument that the treatment of depression may be effective due to the modification of the immune response, is the results of researches carried out with the use of non-steroidal anti-inflammatory drugs (NSAIDs), cytokine inhibitors, polyunsaturated fatty acids or curcumin (Menon and Sudheer 2007). For example, it has been shown that administration of NSAIDs can reduce the symptoms of depression. The cyclooxygenase-2 inhibitor celecoxib turned out to be particularly effective (Köhler et al. 2014). Etanercept, a soluble TNF-α receptor, also was shown to reduce depressive symptoms (Schmidt et al. 2014b). Patients suffering from depression treated with infliximab, an anti-TNF antibody, for longer period of time, also benefited from the therapy in terms of improving their mental state (Raison et al. 2013). Curcumin, a nutrient present in plants of the ginger family, may also contribute to the reduction of depressive symptoms when supplemented with classic antidepressant treatment (Yu et al. 2015), most likely by inhibiting the production of inflammatory mediators, such as prostaglandins, leukotrienes or nitric oxide and increasing in the concentration of biogenic amines in the brain (Kulkarni et al. 2008).

Raison and colleagues who performed randomized clinical trial with above-mentioned infliximab concluded that TNF antagonism does not have generalized efficacy in treatment-resistant depression but may improve depressive symptoms in patients with high baseline inflammatory biomarkers (e.g., high sensitivity C-reactive protein) (Raison et al. 2013). The meta-analysis of randomized, placebo-controlled trials of omega-3 fatty acid treatment of major depressive disorder demonstrated no significant benefit of omega-3 fatty acid administration (Bloch and Hannestad 2012). Authors also concluded that nearly all of the anti-inflammatory treatment efficacy in major depressive disorder observed in the published literature may be attributable to publication bias caused by heterogeneity of the group including somatic or mental comorbidities and pro-inflammatory markers baseline. Lower methodological quality or trials of shorter duration can also affect the final results. Köhler et al. (2014) in their meta-analysis emphasized that identification of subgroups that could benefit from anti-inflammatory treatment is needed especially since NSAIDs treatment is often accompanied by well-known adverse effects.

## Conclusions

According to the assumptions of psychoneuroimmunology, impaired regulation of immune system activity and chronic inflammation may play an important role in the pathogenesis of depression. Furthermore, the results of numerous studies presented in this article show that the mechanism of action of currently used antidepressants is more complex than only theirs influence on the concentration of neurotransmitters in the synaptic cleft. Unfortunately, the results presented here do not always coincide, which may be the result from many different factors, including the heterogeneity of a group of patients diagnosed with a depression, a various mechanism of action of widely understood antidepressants, different doses of drugs administered, treatment period, co-morbidity, polypharmacotherapy or patients’ age. Nevertheless, it seems that modification of the immune system activity may also improve the mental state of patients suffering from affective disorders, which makes the research on the interaction of the immune and nervous systems particularly interesting.
